# Rice farming care as a novel method of green care farm in East Asian context: an implementation research

**DOI:** 10.1186/s12877-021-02181-2

**Published:** 2021-04-09

**Authors:** Chiaki Ura, Tsuyoshi Okamura, Sachiko Yamazaki, Masaya Shimmei, Keisuke Torishima, Akira Eboshida, Yu Kawamuro

**Affiliations:** 1 Research Team for Promoting Independence of the Elderly, Tokyo Metropolitan Institute of Gerontology, 35-2 Sakae-cho, Itabashi-ku, Tokyo, 173-0015 Japan; 2 Bunkyo Gakuin University, 1196 Kamekubo, Fujimino, Saitama, 356-8533 Japan; 3 Den-en Chofu University, 3-4-1 Higashiyurigaoka, Asao-ku, Kawasaki, Kanagawa 215-8542 Japan; 4 Kawamuro Memorial Hospital, 71 Kitashinbo, Joetsu, Niigata, 943-0109 Japan; 5 Graduate School of Biomedical and Health Sciences, Hiroshima University, 1-3-3-2 Kagamiyama, Higashihiroshima, Hiroshima, 739-0046 Japan

**Keywords:** Cognitive function, Dementia, Green care farms, Implementation research, Rice farming care, Well-being

## Abstract

**Background:**

Green care farms, which offer care for people with dementia in a farm setting, have been emerging in the Netherlands. The aim of this study was to 1) implement green care farms which use rice farming in Japan, 2) explore the positive experiences of rice farming care, and 3) compare the effect of rice farming care to that of usual care on well-being and cognitive ability.

**Methods:**

We developed a new method of green care farm in Japan which uses rice farming, a farming that is practiced all over East Asia. The participants were 15 people with dementia (mean age = 75.6 ± 9.8 years) who participated in a one-hour rice farming care program once a week for 25 weeks. We also collected qualitative data on the positive experiences of study participants after the program. As a reference data, we also collected the corresponding data of the usual care group which included 14 people with dementia (mean age = 79.9 ± 5.8 years) who were attending the near-by day-care.

**Results:**

The mean participation rate on the rice farming care group was 72.1%. After the intervention, participants reported experiencing enjoyment and connection during the program. It also changed the staff’s view on dementia. The green care farm group showed a significant improvement in well-being but no significant difference in cognitive function compared to the usual care group.

**Conclusions:**

Green care farms by using rice farming is promising care method which is evidence-based, empowerment-oriented, strengths-based, community-based dementia service, which also delivers meaningful experience for the people with dementia in East Asia.

**Trial registration:**

UMIN, UMIN000025020, Registered 1 April 2017.

**Supplementary Information:**

The online version contains supplementary material available at 10.1186/s12877-021-02181-2.

## Background

A new type of dementia care that is offered on a farm setting, green care farms (GCFs), is emerging from the Netherlands [1]. GCFs programs are an empowerment-oriented, strengths-based, community-based service that aims to improve the quality of life of people with dementia (PWD) [2]. Previous research on residents living on GCFs reported that, compared to usual care, quality of life (QOL) was higher, especially in the areas of positive effect, social relationships, and having something to do [3]; furthermore, caregivers working in GCFs were more positive about the physical environment, activities, and person-centered care [4].

Identifying the most beneficial activities for PWD has been a priority in dementia research, especially given the challenge of aging societies, whereby people worldwide are generally living longer, thus increasing the incidence of dementia. According to Harmer [5], PWD, staff, and carers have different views about what makes activities meaningful; staff and family caregivers consider activities that maintain physical well-being meaningful, whereas PWD find meaning in activities that address their psychological and social needs. According to De Bruins [6], farm activities fit within normal daily life and are considerably different from traditional nursing home activities, which often have an institutional character (e.g., memory training and bingo). In addition, according to Moyle [7], the factors associated with a positive QOL were relationships with family members and other people, the need for control over their lives, and, more importantly, the need to contribute to their communities. According to a qualitative analysis of in-depth interviews with PWD, a key factor in preserving personal dignity is engagement in meaningful activities within the safe and secure environment of the patient’s home [8].

To enable PWD in Japan to perform meaningful activities, we developed the only vernacular GCFs in Japan, which is called rice farming care (RFC) [9–12]. This project began in 2016 and was made available to PWD who visit the day-care center located in the psychiatric hospital, as well as to PWD living in the nearby group home. Historically, people living in psychiatric hospitals and their related institutions are sometimes secluded from their fellow patients, families, friends, and visitors [13]. However, since the launch of this project, we have observed that various people, i.e., family members, volunteers, researchers, and city officers, more frequently visit the hospital and that patient–patient, staff-staff, and patient-staff communication has improved. Thus, while the RFC might have an inclusive effect for otherwise excluded people, the mechanism underlying this effect has not yet been explored. As described elsewhere [10], rice farming has special cultural importance in Japan. For example, rice wine is offered to deities during Japanese rituals; rice plays a crucial role in communal activities, in that to eat rice from the same pan represents strong friendship within a person’s social group; the “land of abundant rice” is often used as a poetic name of ancient Japan; and finally, the emperor himself plants and harvests rice [14]. Rice farming might therefore be a meaningful activity for PWD who can no longer participate in society as before.

The aim of this study was to 1) implement GCFs which use rice farming in Japan, which is common in East Asian environments; 2) explore the positive experiences of PWD who completed the RFC program (qualitative measures); and 3) compare the effect of RFC with that of usual care for well-being and cognitive ability (quantitative measures).

## Methods

We used a realist approach to develop and assess how interventions work in particular contexts to inform future implementation of RFC in real-world environments. We recruited people who visit the day-care center in our hospital and people who live in the group homes next to the hospital. In detail, recruitment was done in outpatient clinic; only those who responded positively to our recruitment participated to our study. Participants were receiving standard care in the day-care center or group homes; we did not replace this care, but offered them additional, year-round, once-a-week, 90-min sessions.

In this setting, randomization was not considered appropriate, i.e. dividing those who wanted to participate into intervention group and control group was not feasible from a realistic viewpoint. At the same time, as an implementation research, to show its effect by comparison was necessary for the future study. Accordingly, we decided to obtain the reference data.

The reference group were recruited in the day-care program or in the group home for PWD, which have been collaborating with our hospital. Reference group’s day-care center or group home was about 30 min’ ride by car, which means that they were not able to participate to our current activity. But, in case our activity expands to other setting, they would be the potential candidates. We adopted a convergent parallel mixed methods design. We collected both quantitative and qualitative data from those who participated in the RFC program. In addition, we collected quantitative data from the reference group. This study was conducted in Niigata prefecture, Japan. The authors confirm that all ongoing and related trials for this intervention are registered.

### Participants

**Fig. 1:**
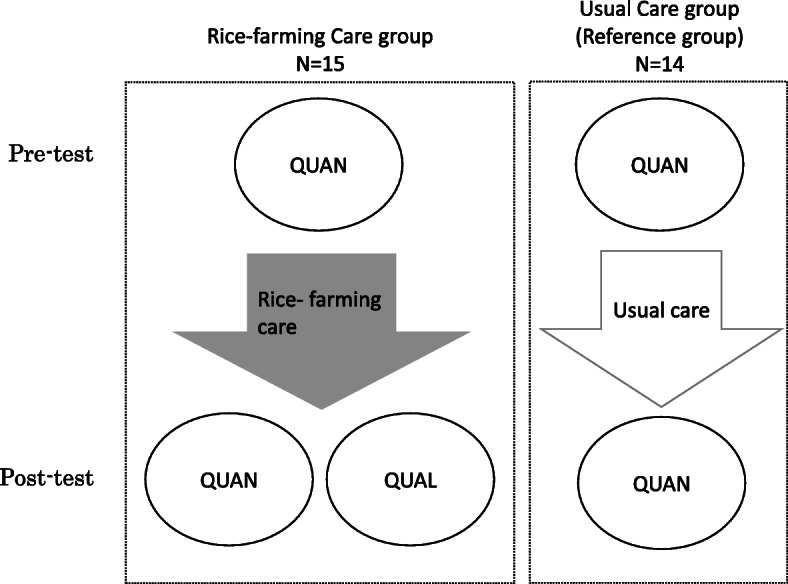
Flowchart of participants. QUAN: Quantitative assessment, QUAL: Qualitative assessment

We compared two groups of community-dwelling older adults with dementia or mild cognitive impairment (MCI), i.e., a RFC group and usual care (UC) group. The RFC group (*n* = 15) participated in our project from April in 2016 to October in 2018, and data from the first year for each participant were included in this study. The UC group (*n* = 14) participated in the day-care program, which is covered by public long-term care insurance, or were living at the group home for PWD. Figure 1 shows the flowchart of the participants.

### Setting

The RFC program was conducted on a rice field (paddy field) and a field of various vegetables, both of which were approximately a 10-min walk from the hospital. Volunteer staff who lived near the hospital, i.e., the president of the neighborhood association and other neighbors, tended both fields outside the activity time as long as they had time.

### Intervention

**Fig. 2:**
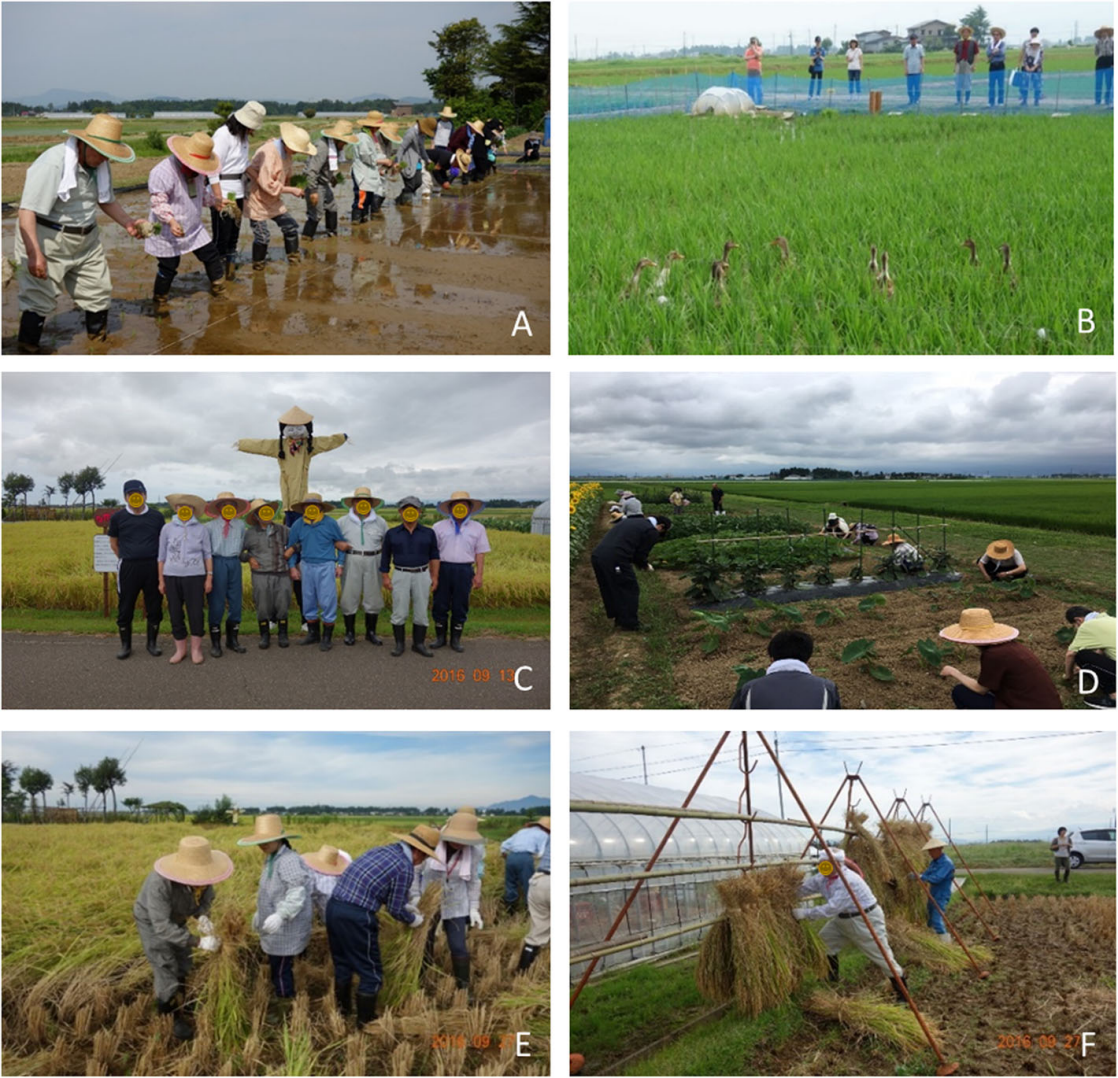
Details of the rice farming care program

The program started with rice planting in May and ended with rice harvesting in October. Every session included physical activity followed by an evaluation meeting, in which PWD, volunteers, and medical staff expressed their thoughts. Details of the RFC program are presented in the Fig. 2.

### Measures

#### Qualitative assessment

We collected qualitative data from the RFC group after the intervention period. The interviews were short and friendly considering that PWD can find it difficult to focus and are not used to speaking with professionals. Semi-structured conversational interviews were implemented by a psychologist and psychiatrist, who asked participants 1) what they thought was good about the rice farming care, and 2) what changes they experienced.

We also collected qualitative data from staff after the intervention period who knew the participants very well. In these conversational interviews, we asked about what changes they observed in participants. (See Supplementary files).

We did not record audio or perform verbatim transcriptions of the interviews, but instead took notes during the interviews.

#### Quantitative assessment

We assessed cognitive ability and mental well-being before and after the intervention period, for both the RFC group and UC group.

We used the Mini-Mental State Examination (MMSE) [15, 16] to assess cognitive impairment. Assessments were conducted by a psychologist or psychiatrist. The MMSE cutoff score for dementia was 23/24.

The Japanese version of the World Health Organization-Five Well-Being Index (WHO-5-J) was used to assess mental well-being [17, 18].

The presence of depressive symptoms was assessed using two question methods [19].

### Analysis

#### Qualitative assessment

The interview notes were read several times to familiarize ourselves with the data. We did not code the interview data because participants’ vocabulary was limited. Instead, we merged themes concerning the good aspects of the farming activity that PWD reported during interviews. From the discourse of the staff, we merged themes concerning changes in patients that were observed by the staff.

#### Quantitative assessment

Effect of intervention within GCF group was assessed by comparing the data before and after the intervention. We analyzed a paired t-test of MMSE and WHO-5-J scores within GCF group.

#### Supplementary analysis

Effect of intervention comparing with reference group was assessed by a two-way analysis of variance that included MMSE and WHO-5-J scores as dependent variables, time, i.e. pre- or post-intervention, as independent variables, and age at the pre-intervention as a covariate to compare with UC group as reference group.

All analyses were conducted using IBM SPSS 23 (IBM Corporation, Armonk, NY, USA).

## Results

### Participant characteristics

Twenty nine participants were analyzed. Descriptive characteristics of the participants are shown in Table 1. There were no between-group differences in age, years of education, cognitive status, well-being, or depression symptoms. However, the RFC group included more males and more participants who had experience of rice farming than the UC group. The mean participation rate on the green care farm group was 72.1%.

**Table 1:** Characteristics of the participants

Mean ± SD	RFC group (*N* = 15)	UC group (*N* = 14)	Total (*N* = 29)	F-value	*P*-value
Age	75.6 ± 9.8	79.9 ± 5.8	77.7 ± 8.3	2.057	0.163
year of education	9.5 ± 2.4	10.6 ± 1.4^a^	10.0 ± 2.1^b^	1.948	0.160
Score of MMSE	20.8 ± 4.3^c^	18.2 ± 7.7^d^	19.6 ± 6.2^e^	1.169	0.290
Score of WHO-5-J	17.5 ± 6.7	17.6 ± 4.8	17.6 ± 5.8	0.003	0.960
Sex (n (%))			Χ^2^	df	*P*-Value
Male	11 (73.4%)	6 (42.9%)	2.773	1	0.096
Female	4 (26.6%)	8 (57.1%)			
Experience of rice-farming (n (%))					
Yes	10 (66.7%)	4 (28.6%)	4.209	1	0.040
No	5 (33.3%)	10 (71.4%)			
Two question (n (%))					
Negative	10 (66.7%)	11 (78.6%)	0.514	1.000	0.474
Positive	5 (33.3%)	3 (21.4%)			

*MMSE* Mini-Mental State Examination, *WHO-5-J* The Japanese version of the World Health Organization-Five Well-Being Index

^a^N of missing value = 1

^b^N of missing value = 1

^c^N of missing value = 1

^d^N of missing value = 1

^e^N of missing value = 2

### Qualitative effects of intervention

The interviews with the participants revealed there to be a subjective efficacy of the rice farming intervention. (See Table 2) The positive aspects of this activity were categorized into two categories, i.e., enjoyment and connection. Table 2-1 shows an example discourse. The subjective changes reported in the interviews with the PWD are shown in Table 2-2, which show the several concrete improvements in daily life and symptoms. Concerning changes that were observed by the staff, four themes were merged, namely 1) the relationship between the PWD and their families; 2) the relationship between the staff members; 3) the relationship between the PWD and staff; 4) the staff’s view on PWD. (See Table 2-3).

**Table 2:** Narratives of the stakeholders

2–1. Positive aspects of the rice farming activity that were reported by the PWD	
**Enjoyment**	
I enjoyed seeing the products.	
I was happy that I could do what I was doing.	
**Connection**	
It was good that I could talk with other people.	
I was looking forward to seeing X (a particular participant) in the session.	
A good thing was that we worked together.	
2–2. Changes observed by the PWD	
I can sleep better.	
I can eat well.	
I talk more frequently.	
I see more new things in life.	
2–3. Changes which were observed by the staff	
1) The relationship between PWD and their families: One participant’s family seldom visited the institution, but came on the day of the harvest, which was surprising to the staff. The family looked satisfied to see the participant enjoying agriculture as before (the participant was a farmer who had owned large rice fields).	
2) The relationship between staff members: One staff member reported that, before the project, discussions were only had between those of the same professions. However, this project enhanced the discussion between doctors, nurses, psychologists, occupational therapists, and students.	
3) The relationship between PWD and staff: One staff member found it difficult to accept the rapid change, i.e., cognitive decline, of one participant, but on seeing the participant’s enjoyment of the activity, was more able to accept these changes.	
4) The staff’s view on PWD: “They had power, and they are living with pride”; “[I realized that participants had once been] young, can walk, and can do their own business”; “At first, I was confused how to communicate with them. I was watching them. After communicating with them, I realized that you don’t need special consideration. You don’t have to talk to a person who seems unwilling to talk. I became relaxed after I got to know them better and became used to how they talk.

**Table 3:** MMSE and WHO-5-J scores in the GCF and UC groups

	RFC group GCF (*N* = 15)				UC group (*N* = 14)				Interaction	
	Mean ± SD of pre	Mean ± SD of post	T-value of paired t-test	*P*-value	Mean ± SD of pre	Mean ± SD of post	T-value of paired t-test	*P*-value	F-value	*P*-value
Score of MMSE	20.8 ± 4.3	21.6 ± 4.2^a^	−1.364	0.196	18.2 ± 7.7	19.0 ± 7.6^b^	1.443	0.175	0.068	0.797
Score of WHO-5-J	17.5 ± 6.7	20.5 ± 3.7	−2.761	0.015	17.6 ± 4.8	16.5 ± 6.7	1.086	0.297	6.472	0.017

*MMSE* Mini-Mental State Examination, *WHO-5-J* The Japanese version of the World Health Organization-Five Well-Being Index

^a^N of missing value = 1

^b^N of missing value = 1

### Quantitative effect of RFC

First, we conducted Kolmogorov-Smirnov test on MMSE and WHO-5-J scores before the intervention; the value was *p* = .200, which indicated a normal distribution of the data. After the intervention, WHO-5-J scores were significantly higher after the intervention, but not cognitive function assessed by MMSE scores. (See Table 3).

### Comparison with the reference group

After the intervention, the WHO-5-J scores were significantly higher in the RFC group than in the UC group (17.5 to 20.5 in the RFC group, 17.6 to 16.5 in the UC group, F = 6.472, *p* = 0.017). MMSE scores after the intervention were not significantly different between the RFC group and UC group (20.8 to 21.6 in the RFC group, 18.2 to 19.0 in the UC group, F = 0.068, *p* = 0.797). (See Table 3).

## Discussion

In this study, we implemented RFC, a newly emerging method that is an empowerment-oriented, strengths-based, and community-based service that aims to improve the QOL in PWD, in a Japanese and East Asian context. We found that well-being of the RCF group was recovered by the intervention, and that RFC group was reported to exert a favorable change on all participants. The main activity of this intervention was rice farming, which is a major aspect of Asian agriculture; our intervention method could therefore be applied in other East Asian countries.

The most important finding of this study was that the intervention was meaningful for PWD. According to Phinney [20], the activities of PWD become meaningful through feelings of pleasure and involvement, a sense of connection and belonging, and a sense of autonomy and self-identity. Indeed, the participants in this study reported feelings of pleasure and involvement and a sense of connection and belonging. Although PWD did not directly mention having a sense of autonomy and self-identity, the staff interviews indicated that RFC increased patients’ sense of autonomy and self-identity; the family of one participant rediscovered his identity through seeing him take part in activities that were similar to his previous work as a farmer, and one staff member rediscovered the strength and pride of PWD.

On the other hand, the green care farm group had more males and more persons previously engaged in rice farming. Therefore, the favorable result may due to returning to familiar and meaningful work for them. Cautious interpretation is essential.

From a clinical standpoint, one of the great changes was that this project shook the old culture of the hospital staff. Given that an impairment in orientation to time is a common symptom of dementia, hospital staff often ask PWD about the date and day of the week to assess cognitive ability. In this project, we often told patients that knowing the date or day of the week did not matter on the farm, and only the season was important. Similarly, dementia is often associated with a decline in instrumental activity of daily living; participants were told that if they could not use the ATM, community living can indeed become more difficult, but this would not impede their farming activity during the intervention. This project was based around person-centered care, whereby PWD are regarded as individuals with unique identities rather than people defined by their symptoms [21, 22].

GCFs has some merits for the rapidly aging societies in which we live; especially given that the number of younger people is simultaneously decreasing. These advantages are as follows: 1) GCFs does not require the construction of new facilities because farms are already there; 2) older farmers can be hired as staff to help with the agricultural processes; 3) GCFs makes use of fallow land effectively. We are planning to use the products in the lunch of a nearby elementary school, which would enhance inter-generation communication.

This study has several limitations. First, it was conducted in only one area in Japan and the number of participants was small. A confirmation study at multiple sites would therefore be necessary for generalization of the results. Second, we did not record the audio or produce verbatim transcriptions in our qualitative analysis. Additionally, the vocabulary of PWD was limited, and so video recordings and visual analysis might help to overcome this limitation. Third, we could not control the activity of the reference group, such as frequency, intensity, and length. This was beyond our capacity. Forth, intervention group and reference group might not be homogeneous, meaning that selection bias is inevitable; the intervention group had more males and more persons previously engaged in rice farming.

## Conclusions

Green care farms by using rice farming is promising care method which is evidence-based, empowerment-oriented, strengths-based, community-based dementia service, which also delivers meaningful experience for the people with dementia in East Asia. Although it might be a challenge in a real world setting, further research using the more empirical methodology such as randomization is necessary for the further implementation.

## Supplementary Information

**Additional file 1.**

## Data Availability

The datasets used and/or analyzed during the current study are available from the corresponding author on reasonable request.

## Abbreviations

GCFs: Green care farms
PWD: People with dementia
QOL: Quality of life
RFC: Rice farming care
UC: Usual care
MMSE: Mini-Mental State Examination
WHO-5-J: The Japanese version of the World Health Organization-Five Well-Being Index
