# Supplementary material for: Rice farming care as a novel method of green care farm in East Asian context: an implementation research
Source: BMC Geriatr. 2021 Apr 9;21:237. doi: 10.1186/s12877-021-02181-2 (PMC8034120; doi:10.1186/s12877-021-02181-2)
Supplement: Supplementary file 1 — Additional file 1. [file 12877_2021_2181_MOESM1_ESM.docx]

Interview items

Subjective: To participants

1. What do you think was good about the rice farming care?
2. What changes did you experience?

Objective: To staff

1. What changes did you observe in participants?

Notice

1. The interviews with participants should be short and friendly. The reason is that people with dementia might find it difficult to focus and be not used to speaking with professionals.
2. The interviews should be conducted by a psychologist or psychiatrist.
